# Novel pulmonary vascular imaging signs in COVID-19: pathophysiology, significance and management

**DOI:** 10.1259/bjro.20210001

**Published:** 2021-07-05

**Authors:** Arshed Hussain Parry, Abdul Haseeb Wani

**Affiliations:** 1 Department of Radiodiagnosis, Sher-i-Kashmir Institute of Medical Sciences, Srinagar, Jammu & Kashmir, India; 2 Department of Radiodiagnosis, Government Medical College, Srinagar, Jammu & Kashmir, India

## Abstract

A growing body of evidence points to the frequent involvement of pulmonary microvessels in COVID-19 which was recognized first on CT, and subsequently demonstrated by clinical and pathological studies. Microvasculopathy occurring chiefly from endothelial and pericyte damage with resultant disruption of immune, thrombotic and renin–angiotensin–aldosterone balance leads to a constellation of clinical and biochemical derangements. Exploration of potential therapies directed at normalizing the vascular health can prove a major boon in the treatment of COVID-19.

## Introduction

The ongoing pandemic of coronavirus disease 19 (COVID-19) is a highly contagious viral disease caused by severe acute respiratory syndrome coronavirus-2 (SARS-CoV-2). The disease is known to have systemic manifestations with lungs being the primary target organ. The widespread use of chest CT has led to the recognition of hitherto unreported imaging signs not described earlier in infectious disease settings. These novel imaging signs pertaining to small pulmonary vessels are observed frequently in addition to the findings of pneumonia like ground glass opacification (GGO) or consolidations in the affected individuals.

## Novel imaging signs

**Figure 1.**
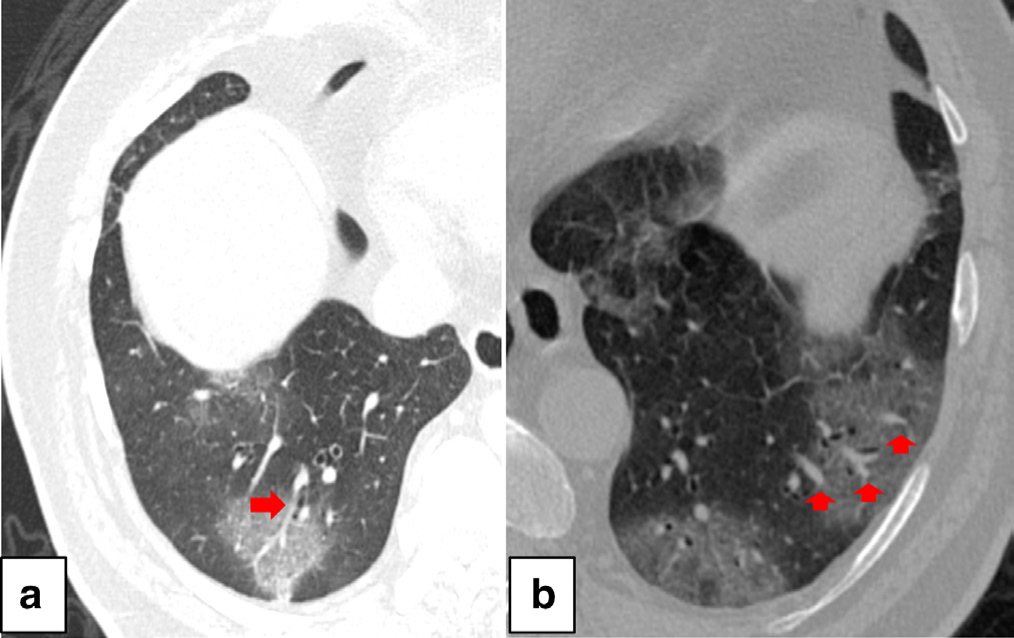
Axial chest CT images in lung window settings obtained through the lower lobes in two COVID-19 patients showing multifocal GGOs with intralesional vessel thinning (red arrow in A) and VES (red arrows in B). GGO, ground glass opacity; VES, vascular enlargement sign.

**Figure 2.**
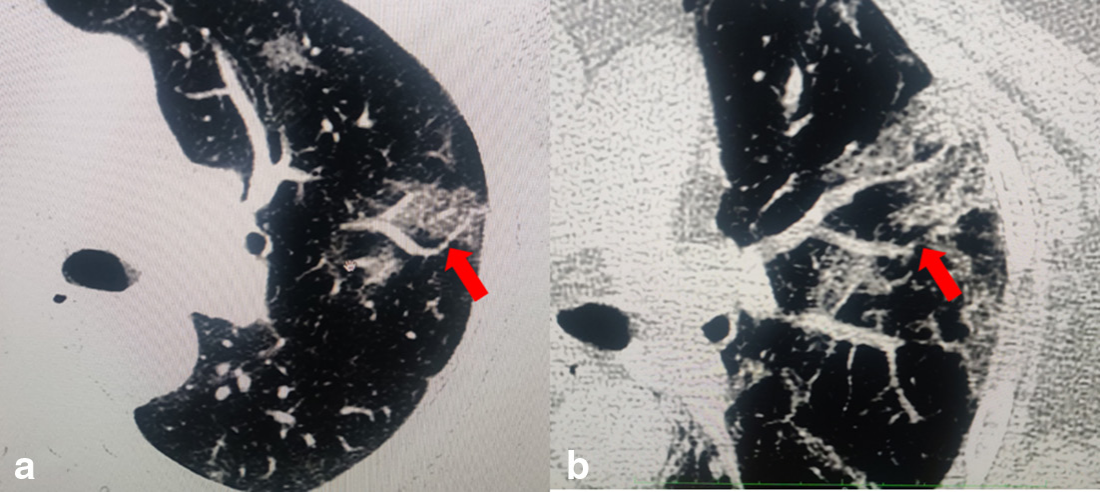
Axial chest CT images in lung window settings obtained through the upper lobes in two COVID-19 patients showing multifocal GGOs with focal concentric narrowing of segmental pulmonary vessel (vascular knuckle sign) (red arrow in A) and deviation of vessel from the expected course (vessel angulation sign) (again denoted by red arrow in A) and obscuration of segmental pulmonary vessel near GGO producing invisible vessel sign (red arrows in B). GGO, ground glass opacity.

A common imaging sign was vascular enlargement sign (VES). VES has been reported in approximately two-thirds of COVID-19 pneumonia.^1,2^ VES was variably defined on CT as a subjective enlargement of small pulmonary vessels compared to the contralateral lung or objectively by vessel diameter of more than 3 mm in or around the pulmonary lesions.^2,3^ Vessel enlargement has been noted not only in the parenchymal opacities but also in the surrounding normal lung reaching as far as the pleural surface (Figure 1). Subsequently, additional vascular signs like vascular thinning, invisible vessel sign, vessel wall irregularity and knuckle like vascular angulation were reported.^4^ Vessel thinning has been used to describe >20% reduction of vessel diameter within the pulmonary lesion. Invisible vessel sign represents complete obscuration of vessel within a GGO. A bent of >30 degree of vessel wall from its normal expected course is referred to as vascular angulation. Vascular knuckle has been used to define segmental concentric narrowing of the vessel (Figure 2). Vessel wall irregularity in a pulmonary lesion has also been reported.^4^ Acute fibrinous and organizing pneumonia (AFOP), a histological variant of organizing pneumonia, characterized by formation of fibrin-balls in the alveoli without hyaline membrane formation, has also been described infrequently in COVID-19. Although variable imaging findings in AFOP have been described, patients who experience a rapid respiratory deterioration demonstrate imaging findings similar to diffuse alveolar damage (DAD), with diffuse but basilar-predominant consolidation and GGOs. The patients with a more subacute course may exhibit similar imaging findings of cryptogenic organizing pneumonia with focal or diffuse parenchymal abnormalities.^5^

## Pathophysiology

Three mechanisms were proposed to account for VES. Vasodilatation caused by the release of proinflammatory cytokines, infection-induced pulmonary vasculitis or *in-situ* microthrombosis of segmental or subsegmental pulmonary arteries were believed to be responsible for VES.^6,7^ Vascular thinning or invisibility is believed to be caused by reflex vasoconstriction due to ensuing hypoxemia. Vascular angulation and knuckle deformity is purportedly caused by the fibrotic response during the subacute-chronic phase of disease.^4^ These dramatic vascular changes reported with high frequency garnered wide attention, however, a precise understanding of underlying pathophysiological mechanisms was lacking. So, it was left to the autopsy studies to adjudicate the final decision on the exact pathogenesis of these vascular changes.

Autopsy studies have consistently revealed evidence of diffuse alveolar damage, vascular inflammation and microthrombi formation in COVID-19 decedents. In a multicentre autopsy study, Borczuk et.al^8^ reported that microthrombi were present in 84% of cases apart from large vessel thrombi in 42%. These microthrombi, composed of platelets and fibrin, were detected in small arteries (<1 mm) and in pulmonary capillaries. These microthrombi were seen despite patients receiving anticoagulation therapy. Ackerman et. al^9^ in an autopsy study demonstrated that COVID-19 is characterized by severe endothelialitis involving small pulmonary vessels (diameter 1–2 mm) with disruption of intercellular junctions alongside widespread microthrombosis with evidence of intussusceptive neoangiogensis. Pulmonary microthrombosis was nine times more prevalent in COVID-19 decedents compared to the influenza (*p* < 0.001).

A healthy vascular endothelium is pivotal to the maintenance of vascular permeability, inflammatory equilibrium, hemodynamic stability, immune competence and balance between thrombotic and antithrombotic pathways. Vascular endothelial cells express angiotensin converting enzyme 2 (ACE-2) which is the target receptor of SARS-CoV-2. Endothelial cell damage caused by viral particles incites profound vascular inflammation and precipitates thrombosis. Microvasculopathy consisting of severe vascular inflammation with thrombosis seems to represent the imaging correlate of VES. Apart from direct endothelial damage, loss of pericysts is also incriminated in pulmonary microvasculopathy. Pericysts are a unique type of perivascular cell which are indispensable to the integrity of microvessels. These cells exhibit abundant ACE-2, the entry receptor for SARS-CoV-2. ACE-2-mediated COVID-19-endotheliitis also explains the microcirculatory dysfunction in different organ vascular beds and their resultant clinical consequences. ACE-2 receptors, which are expressed abundantly in lungs, intestinal enterocytes and nerve cells also accounts for the direct damage to these organs.

The microvasculopathy resulting from severe vessel wall inflammation with formation of microthrombi significantly contributes to hypoxemia in COVID-19 patients. Initially, it was thought that acute lung injury mediated by virus-induced pneumocyte damage is responsible for hypoxemia. However, with the advent of time it was recognized that severe refractory hypoxemia in severe cases cannot be solely attributed to viral pneumonia as the degree of hypoxemia is inconsistent with the amount of reduction in lung function or pulmonary compliance. This led to the recognition of vascular dysfunction as an important contributor to morbidity and mortality in COVID-19.^10^

## Significance

These pulmonary vascular signs have both diagnostic and prognostic significance when recognized on CT. Presence of VES is a valuable ancillary finding that could potentially help in distinguishing between COVID-19 pneumonia and other causes of acute lung injury patterns.VES was reported to be specific for COVID-19 pneumonia and was supposed to have a diagnostic value in discriminating COVID-19 pneumonia from infectious mimics like influenza. Prognostically, presence of VES and other microvascular changes are associated with elevated inflammatory markers and d-dimer levels and are harbingers of a poor prognosis.^4,11^

## Diagnosis

It is challenging to find the direct evidence of microvascular obstruction on computed tomographic pulmonary angiography (CTPA). CTPA which is the standard diagnostic tool to detect pulmonary macroembolism unfortunately does not demonstrate microvascular obstruction as it beyond the thresholds of the currently available CT technology. Dual energy computed tomography (DECT) is a versatile modality capable of demonstrating microvascular abnormalities *in vivo*. DECT by utilizing the principle of tissue iodine distribution generates color-coded perfusion maps, and any perfusion abnormality manifests as a perfusion defect. DECT perfusion imaging has demonstrated striking perfusion abnormalities in COVID-19 patients comprising of regional oligemia overlapping or corresponding to the areas of pulmonary opacification. Perfusion abnormalities were observed in 100% cases that required mechanical ventilation or demised and in 95% cases who did not require ventilation. All patients were seen to demonstrate elevated inflammatory and prothrombotic biomarkers. It is noteworthy to mention that hypoperfusion abnormalities were observed despite patients receiving anticoagulation. Regional areas of hyperemia matching pulmonary opacities may be observed in the first week of illness. A low ventilation/perfusion ratio in first week may be due to reduced ventilation (secondary to pneumonia) and normal or increased perfusion (due to loss of normal hypoxia vasoconstriction mechanism).^2,10,11^

## Management

From the management point of view, tackling pulmonary vascular inflammation in conjunction with anticoagulation is essential to improve the outcome of these patients. Strategies aimed at muting the hyperinflammatory drive, especially before entering the severe stage of disease, could change the trajectory of illness. Similarly, amelioration of vascular dysfunction by reducing angiotensin-II-induced endothelial inflammation could be potentially beneficial. Glucocorticoids (dexamethasone) and interleukin-6 inhibitors (tocilizumab and sarilumab) quell the hyperimmune response and have shown beneficial results in severely ill patients.^12^ But the beneficial effects of tocilizumab have not been reproduced by all the published studies. Currently, clinical trials are underway to explore the role of bevacizumab (anti-VEGF monoclonal antibody) and razuprotafib (novel Tie two activator) in ameliorating endothelial dysfunction in COVID-19 patients.^13^ We await the results of these trials with hope.

In conclusion, a comprehensive understanding of the pathophysiology underpinning the frequent and dramatic microvascular events in COVID-19 would stimulate the search for new therapeutic targets.
